# Design and Numerical Analysis of an Infrared Cassegrain Telescope Based on Reflective Metasurfaces

**DOI:** 10.3390/nano11112904

**Published:** 2021-10-29

**Authors:** Song Yue, Zhe Zhang, Kunpeng Zhang, Huifang Guo, Ran Wang, Tonghui Dou, Dongliang Zhang, Lianqing Zhu, Haining Yang, Zichen Zhang

**Affiliations:** 1 Microelectronics Instruments and Equipment R&D Center, Institute of Microelectronics, Chinese Academy of Sciences, 3 Beitucheng West Road, Beijing 100029, China; yuesong@ime.ac.cn (S.Y.); zhangzhe1@ime.ac.cn (Z.Z.); zhangkunpeng@ime.ac.cn (K.Z.); guohuifang@ime.ac.cn (H.G.); wangran@ime.ac.cn (R.W.); doutonghui@ime.ac.cn (T.D.); 2 School of Microelectronics, University of Chinese Academy of Sciences, No. 19(A) Yuquan Road, Beijing 100049, China; 3 Key Laboratory of the Ministry of Education for Optoelectronic Measurement Technology and Instrument, Beijing Information Science & Technology University, 12 Xiaoying East Road, Beijing 100192, China; zdl_photonics@bistu.edu.cn (D.Z.); zhulianqing@sina.com (L.Z.); 4 Department of Electronic Science and Engineering, Southeast University, 2 Sipailou, Nanjing 210018, China

**Keywords:** reflective metasurface, Cassegrain telescope, silicon dioxide, aluminum, fabrication error

## Abstract

Reflective imaging systems such as Cassegrain-type telescopes are widely utilized in astronomical observations. However, curved mirrors in traditional Cassegrain telescopes unavoidably make the imaging system bulky and costly. Recent developments in the field of metasurfaces provide an alternative way to construct optical systems, possessing the potential to make the whole system flat, compact and lightweight. In this work, we propose a design for a miniaturized Cassegrain telescope by replacing the curved primary and secondary mirrors with flat and ultrathin metasurfaces. The meta-atoms, consisting of SiO_2_ stripes on an Al film, provide high reflectance (>95%) and a complete phase coverage of 0~2π at the operational wavelength of 4 μm. The optical functionality of the metasurface Cassegrain telescope built with these meta-atoms was confirmed and studied with numerical simulations. Moreover, fabrication errors were mimicked by introducing random width errors to each meta-atom; their influence on the optical performance of the metasurface device was studied numerically. The concept of the metasurface Cassegrain telescope operating in the infrared wavelength range can be extended to terahertz (THz), microwave and even radio frequencies for real-world applications, where metasurfaces with a large aperture size are more easily obtained.

## 1. Introduction

Refractive and reflective optical components are the two main basic categories of modern optical systems. Compared to the essential roles played by lenses in refractive optics, optical mirrors receive less attention in modern optics research. On the other hand, reflective optical components are widely used and even irreplaceable in many optical systems, such as cameras, microscopes and other imaging systems, especially in modern telescopes and infrared systems. The telescope is a kind of typical optical system which is especially important in the field of astronomical observations. Many modern optical telescopes are based on reflective optics, such as the Hubble Space telescope, the Keck Telescope and the Very Large Telescope [1,2], etc. To facilitate convenient imaging functionality, it is desired that the object and image lie on opposite sides of the imaging system. While a single refractive lens can achieve this, representing the simplest imaging system, a single reflective mirror cannot. Thus, a conjunction of two mirrors is required to construct an imaging system based on reflective optical components. Among various configurations, conventional Cassegrain systems composed of two curved mirrors, called primary and secondary mirrors, are commonly seen in focusing and imaging systems, possessing the advantage of being able to shorten the system length compared to the single-lens system. Reflective optical systems rely on the precise surface profiles of the curved mirror to realize their optical functionality [3], which unavoidably renders the whole system cumbersome and costly. Specifically, with increasing aperture size and decreasing working-wavelength, satisfaction with respect to the requirement of surface figure errors (<λ for commercial, <1/4 λ for precision and <1/20 λ for high-precision instruments) becomes increasingly challenging, even with state-of-the-art fabrication techniques [1,4,5].

In 2011, a revolutionary category of optical devices called metasurface emerged [6,7]. Composed of arrays of subwavelength entities called meta-atoms, metasurfaces can flexibly manipulate multiple degrees of freedom of light, such as its amplitude, phase, polarization and wave vector, etc. Compared to traditional refractive and reflective optical devices that rely on the propagation/reflection of light through/on curved surfaces, metasurfaces manipulate light through a flat interface with elaborately introduced phase discontinuity. Being flat and ultrathin, metasurfaces can effectively reduce the size and weight of many optical systems, attracting increasing research interest in recent years. Researchers have demonstrated a plethora of ultrathin and flat optical devices based on metasurfaces, including metalens [8,9], beam deflectors [10,11], meta-holograms [12,13], color filters [14,15], optical vortex generators [16,17], nonlinear [18,19] and even quantum [20,21] optical devices, etc. Among them, Li et al. demonstrated for the first time a Cassegrain telescope based on two flat, ultrathin metasurfaces operating in the infrared wavelength range [22], opening up a new avenue to circumvent difficulties faced by reflective optics with curved surfaces. However, that work utilizes geometric Pancharatnam–Berry (P–B) phase as the phase manipulation mechanism and employs SiO_2_ and Au as the constituent materials to construct the metasurface. Therefore, there is still room to improve the performance of metasurface Cassegrain telescopes considering the following facts. First, geometric phase requires incident light to be circularly polarized; thus, the limitation on the polarization state of incoming light reduces the applicability of the metasurface telescope. Secondly, the constituent material Au has limited working wavelength range, i.e., Au is quite absorptive for wavelengths below 500 nm due to inter-band transitions, so it is not suitable for short-wavelength applications. Moreover, Au is expensive and not compatible with current complementary metal-oxide semiconductor (CMOS) technology, hindering its potential for large-scale applications.

To tackle the abovementioned challenges, we propose in this work another type of metasurface Cassegrain telescope based on a propagation phase manipulation mechanism, with SiO_2_ and Al as the constituent materials. The propagation phase manipulates the transmission/reflection phase by varying the lateral size of meta-atoms and can, in principle, achieve polarization-independent phase modulation if meta-atoms with high geometric symmetry are used, such as square- or circle-shaped pillars [23]. Moreover, the SiO_2_ plus Al material combination has quite low optical absorption from deep-ultraviolet (DUV, ~190 nm) all the way to the mid-wave infrared wavelength range (MWIR, ~7 μm), possessing the potential to serve as efficient building blocks of metasurface devices across a very broad wavelength range. Previously, we proposed and numerically demonstrated a kind of reflective metalens based on the SiO_2_ plus Al combination working in the DUV to visible wavelength range [24]. In this work, a reflective metasurface Cassegrain telescope was designed for the operational wavelength of 4 μm—an important infrared atmospheric window wavelength. The optical performance of the metasurface Cassegrain telescope was studied numerically using the finite element method. Moreover, since fabrication errors usually reduce the optical performance of metasurface devices [25,26], and studies on this topic are relatively rare, we numerically studied the influence of random size errors of meta-atoms on the overall optical performance of metasurface Cassegrain telescopes. We found that a random size error of up to 25% is allowable for the metasurface device to function correctly—a value much greater than that of traditional Cassegrain telescopes based on curved mirrors [1,4,5]. The metasurface Cassegrain telescope proposed and numerically demonstrated in this work provides an alternative design towards ultrathin, flat optical imaging systems, which can be easily scaled-up for real-world applications if we switch from infrared to the terahertz (THz) or micro-wave regimes, where metasurface devices with large apertures are more easily obtained.

## 2. Design and Simulation Methods

**Figure 1:**
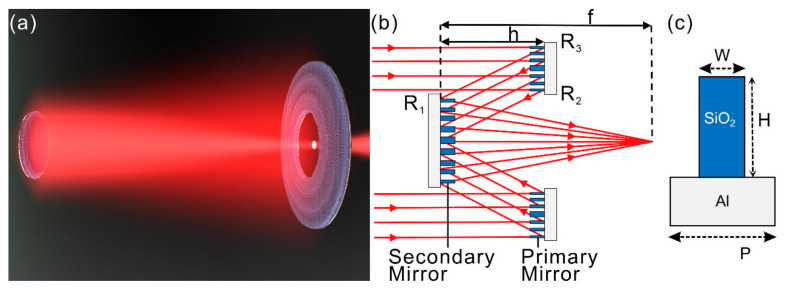
(**a**) Schematic illustration of a Cassegrain telescope based on reflective metasurfaces. (**b**) Schematic drawing of the optical path as well as the design parameters of the metasurface Cassegrain telescope, including R_1_, R_2_, R_3_, h and f. (**c**) Meta-atom of the metasurface, which consists of an SiO_2_ stripe on top of an Al mirror. Period, width and height of SiO_2_ stripe are labeled as P, W and H, respectively.

A schematic illustration of the metasurface Cassegrain telescope based on reflective metasurface mirrors is displayed in Figure 1a,b. A classical Cassegrain telescope consists of curved primary and secondary mirrors, both of which were replaced with ultrathin, flat metasurfaces here. Figure 1b schematically depicts the optical path as well as the design parameters of a metasurface Cassegrain telescope, including the radius of the secondary mirror (R_1_), the inner and outer radius of the primary mirror (R_2_ and R_3_, respectively), the offset between the two mirrors (h) and focal length of the system (f). Similar to a classical Cassegrain telescope, the primary mirror of the metasurface telescope focuses light towards the secondary mirror, which defocuses incoming converging light to the system focus behind the exit pupil of the primary mirror. Unlike curved mirrors in a classical Cassegrain telescope, each mirror of the metasurface telescope is constructed with meta-atoms, as shown in Figure 1c. In this work, we chose an SiO_2_ stripe on top of an optically thick Al film (thickness ≥ 200 nm) as the basic building block because both materials are abundant and CMOS-compatible, with superior optical properties in the DUV to MWIR wavelength range [24]. Considering that the full-wave simulation of a three-dimensional (3D) metasurface Cassegrain telescope requires a huge amount of memory, we decided to simplify our model to a two-dimensional (2D) case due to limited computation resources. The difference is, in a 3D model, if one uses a square- or circle-shaped SiO_2_ pillar on top of an Al film, the metasurface device will obtain the property of polarization-independence [27]. On the contrary, in the case of 2D models, the polarization of incident light should be transverse magnetic (TM) polarized. However, discussions on the focusing properties of the metasurface telescope and the influence of fabrication errors are still valid and can be extended to the 3D case. Therefore, all simulations were done with 2D models for the following. The period of the meta-atom is P, and the width and height of the SiO_2_ stripe are W and H, respectively. With an appropriate period and height, meta-atoms with different widths can offer a phase coverage of 0~2π, with high reflection, serving as suitable building blocks to construct metasurface devices.

Numerical analysis of the optical property of the meta-atoms and metasurface devices was carried out using COMSOL Multiphysics—a commercial software based on the finite element method (FEM). In our simulations, we constructed the metasurface Cassegrain telescope with a home-written MatLab code, and then surrounded the whole simulation domains with perfect matching layers (PML). All outer boundaries of the PML domains were set as scattering boundary conditions (SBC). In this way, due to the excellent absorption capacity of the PML + SBC combination, any scattered light that is incident on the PML region will be absorbed and will not affect the electromagnetic field distribution inside the simulation domain. Three inner boundaries were set as the launching and listening ports, respectively. The reflectance and reflection phases of meta-atoms with different geometric parameters were extracted and studied.

Metasurfaces rely on arrays of meta-atoms to reproduce the required local phase change of the desired optical device. To achieve that, the required phase profile of a given optical component is discretized with subwavelength resolution. On the one hand, the period of meta-atoms should be smaller than the corresponding operational wavelength, otherwise the higher-order grating diffraction mode emerges, which reduces the efficiency of metasurfaces [28]. On the other hand, the period of meta-atoms should not be too small, otherwise there will not be enough space for the size of the meta-atoms to vary, rendering the phase coverage range to be less than 2π.

**Figure 2:**
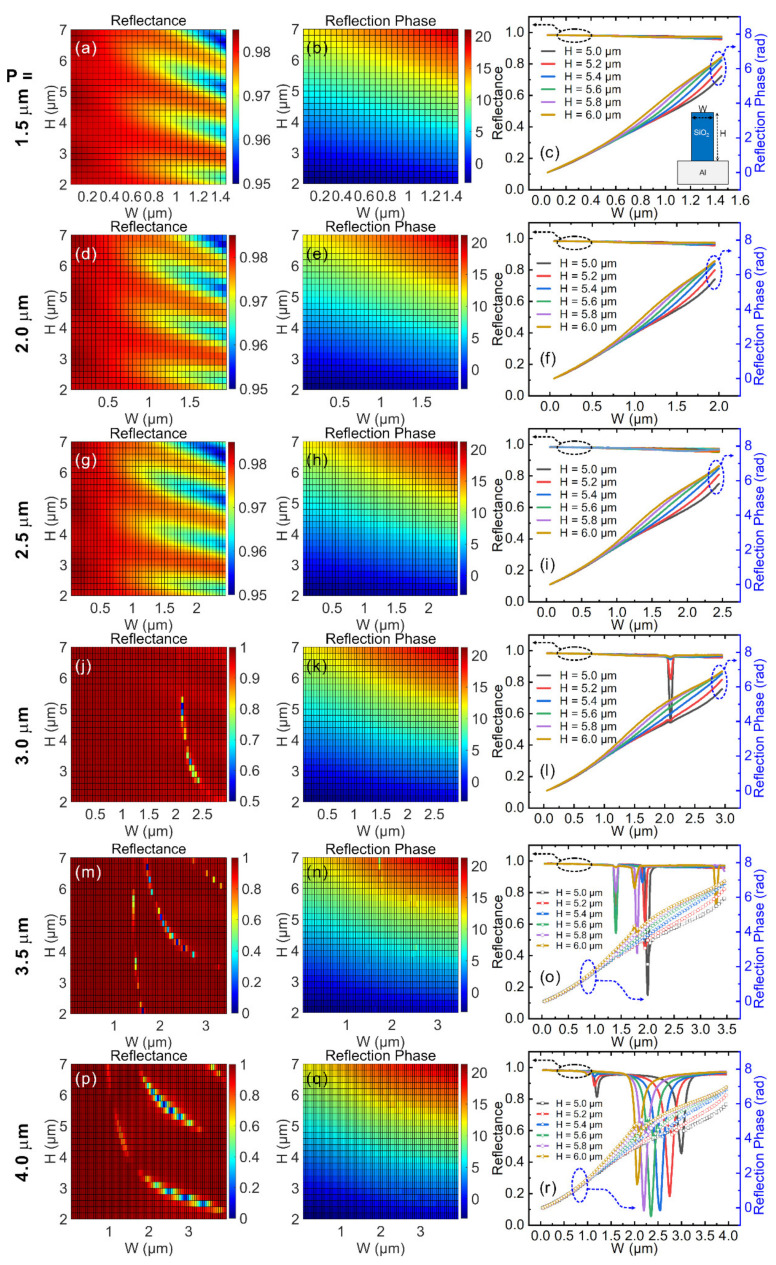
Simulation data for parameter sweep. The period (P) of meta-atoms changed from 1.5 to 4.0 μm in steps of 0.5 μm. For each period, the width (W) of SiO_2_ stripes changed from 0.05 μm to P—0.05 μm in steps of 0.05 μm, and height (H) of SiO_2_ stripes changed from 2.0 μm to 7.0 μm in steps of 0.2 μm. Left column (**a**,**d**,**g**,**j**,**m**,**p**): color map of reflectance with various W and H values under different value of P; middle column (**b**,**e**,**h**,**k**,**n**,**q**): color map of reflection phase; right column (**c**,**f**,**i**,**l**,**o**,**r**): curves of reflectance and reflection phase for an easier selection of appropriate W and H values.

Based on the above concerns, we conducted a large-scale parameter sweep to find the optimal values of period (P), width (W) and height (H) of the meta-atoms; the results are displayed in Figure 2. The period (P) of the meta-atom changes from 1.5 to 4.0 μm in steps of 0.5 μm. For each value of P, the W and H parameters were swept to obtain a map of the reflectance and reflection phases (left and middle columns of Figure 2). The reflectance and reflection phases were replotted in the right column to facilitate the easy selection of optimal parameter combinations. The selection criterion was that the meta-atoms selected must provide a complete phase coverage of 0~2π while maintaining high reflectance. As seen from Figure 2j,m,p, when the period is too large, e.g., P ≥ 3.0 μm, some dips with low reflectance appear, which correspond to the Mie-resonances of light supported by the SiO_2_ stripes [29]. Thus, meta-atoms with a period greater than 3.0 μm can be excluded because meta-atoms with low reflectance cannot be used, otherwise the overall efficiency of the metasurface device is low. When the period was smaller than 3.0 μm, it was found that all meta-atoms simulated maintained high reflectance (>95%) due to the low absorption property of the SiO_2_ plus Al combination at 4 μm wavelength, and the phase change range covered 2π with appropriate height. In principle, one can also choose P = 2.0 or 2.5 μm as the period of meta-atoms. However, these period values are not small enough to catch the fast-varying phase profile of the Cassegrain telescope in certain cases. In order to obtain a high-resolution reproduction of the required phase profile of the Cassegrain telescope, we chose P = 1.5 μm as the optimized period of the meta-atoms, which is a relatively small period. Then, we scanned the width (W) from 0.05 to 1.45 μm and the height (H) from 2 to 7 μm. As shown in Figure 2b, different phase coverage ranges were achieved for meta-atoms with different heights. To facilitate a straightforward comparison, we replotted reflectance and reflection phases within the height range from 5 to 6 μm, shown in Figure 2c. As can be seen from Figure 2c, all meta-atoms within this height range demonstrated high reflectance (>95%), and their phase coverage was close to 2π. A closer look at the phase data reveals that meta-atoms with heights of 5.0 and 5.2 μm covered a phase change range of 5.59 and 6.12 radians, respectively (slightly smaller than 2π). For the height of 5.4 μm, the phase coverage range reached 6.49 radians (exceeding 2π). Higher SiO_2_ stripes provide an even larger range of phase coverage, which is not necessary since a smaller height is preferable from the experimental fabrication point of view. Therefore, we can adopt the optimal height value of H = 5.4 μm. So far, we can set the parameters as P = 1.5 μm and H = 5.4 μm. When the width (W) of the SiO_2_ stripes changed from 0.05 to 1.45 μm, all the meta-atoms maintained a reflectance higher than 95% and covered a phase change range over 2π, serving as good building blocks to construct metasurface devices.

To construct a metasurface device, the required phase profile of the corresponding optical component should first be obtained. For a classical Cassegrain telescope, the required phase distribution on the primary (Φ_P_) and secondary (Φ_S_) mirror can be obtained through the following formulas [22]:

(1) $$\Phi_{p}=\frac{2\pi n}{\lambda }\int_{0}^{{|r}_{p}|}\frac{r_{2}{-r}_{1}}{\sqrt{{{(r}_{2}{-r}_{1})}^{2}{+h}^{2}}}{\mathrm{dr}}_{2},\;\;\;\;\;\;R_{2}\leq {|r}_{p}|\leq R_{3}$$

(2) $$\Phi_{s}=\frac{2\pi n}{\lambda }\int_{0}^{{|r}_{s}|}\left[\frac{r_{1}}{\sqrt{r_{1}^{2}{+f}^{2}}}-\frac{r_{2}{-r}_{1}}{\sqrt{{{(r}_{2}{-r}_{1})}^{2}{+h}^{2}}}\right]{\mathrm{dr}}_{1},\;\;\;0\leq {|r}_{s}|\leq R_{1}$$

**Figure A1:**
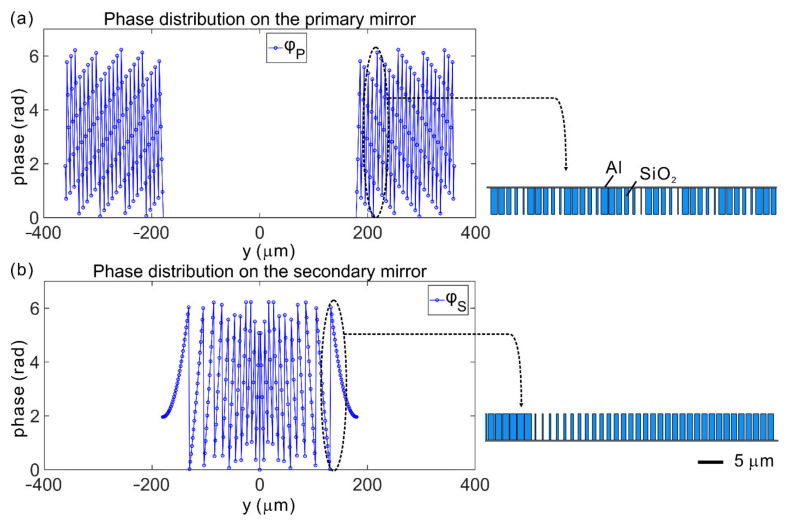
Phase distribution on (**a**) primary and (**b**) secondary mirrors of a typical metasurface Cassegrain telescope.

Here, R_1_, R_2_ and R_3_ represent the radius of the secondary mirror and the inner and outer radius of the primary mirror, respectively (see Figure 1b). h is the offset distance between the primary and secondary mirrors, and f is the focal lens of the telescope system. n is the refractive index of the surrounding medium of the metasurface, which is 1 in this work since the metasurface telescope operates in air. λ is the targeted operational wavelength, which is 4 μm in this work. r_2_ and r_1_ are the radial coordinates of a moving point from the center to the edge of the metasurface, and |r_p_| and |r_s_| are the upper integration limits used to calculate the local phase on the primary and secondary mirror, respectively (see also the Supplementary Information of the reference [30]). Using the above formula, the required phase distribution on the primary and secondary mirrors of the metasurface Cassegrain telescope can be calculated with a home-written code. The required phase distribution on primary and secondary mirrors of a typical metasurface Cassegrain telescope with the following design parameters is plotted in Figure A1 of Appendix A: R_1_ = R_2_ = 180 μm, R_3_ = 360 μm, h = f = 300 μm. With the phase profiles at hand, one then matches the required phase of the metasurface device and the phase change provided by meta-atoms and places at each position the corresponding meta-atom. In this way, a layout for the metasurface device is obtained, which can be used for subsequent numerical simulations and/or fabrications.

## 3. Results, Analysis and Discussions

**Figure 3:**
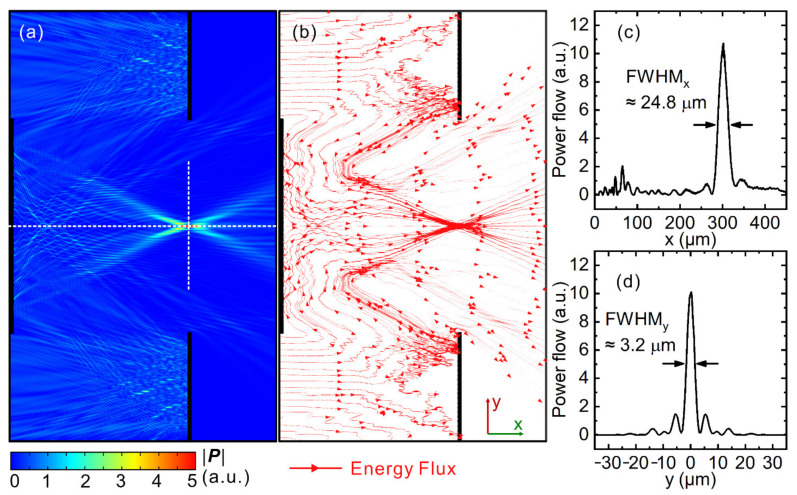
(**a**) Energy flux of a typical metasurface Cassegrain telescope with design parameters R_1_ = R_2_ = 180 μm, R_3_ = 360 μm and h = f = 300 μm. (**b**) Poynting vectors showing that the metasurface conjunction redirects incident energy flux in the way a Cassegrain telescope does. (**c**,**d**) Line-cut plots of energy flux along the white dashed lines in (**a**) along (**c**) x- and (**d**) y-direction, respectively.

A metasurface Cassegrain telescope with design parameters R_1_ = R_2_ = 180 μm, R_3_ = 360 μm, and h = f = 300 μm was constructed and studied numerically; the results are shown in Figure 3. As evident from Figure 3a, light incident on the primary mirror is first deflected/focused towards the secondary mirror and then refocuses to the system focus. At the exit pupil of the telescope, a clear focus can be observed. Obviously, the functionality of a Cassegrain-type telescope is realized with flat metasurfaces. The energy flux depicted in Figure 3b more clearly shows the direction of energy flux, which further confirms the function of the metasurface conjunction. Line-cut plots along the x- and y-directions of the focus are plotted in Figure 3c,d, and the full width half maximum (FWHM) of the focus is 24.8 and 3.2 μm along the x- and y-directions, respectively. A FWHM value in the y-direction of 3.2 μm implies that the metasurface Cassegrain telescope can achieve sub-wavelength focusing, given that the designed operational wavelength is 4 μm. In traditional Cassegrain telescopes with curved mirrors, the focal position is usually behind the exit pupil of its primary mirror for ease of use. In this work, to fully utilize metasurface optical devices’ advantage of being flat and ultrathin, we purposely set the focal position exactly at the exit pupil of the telescope system. In this way, a flat CMOS detector array can be mounted at the back plane of the primary mirror, effectively reducing the length of the whole system along its optical axis and increasing the compactness of the system.

**Figure 4:**
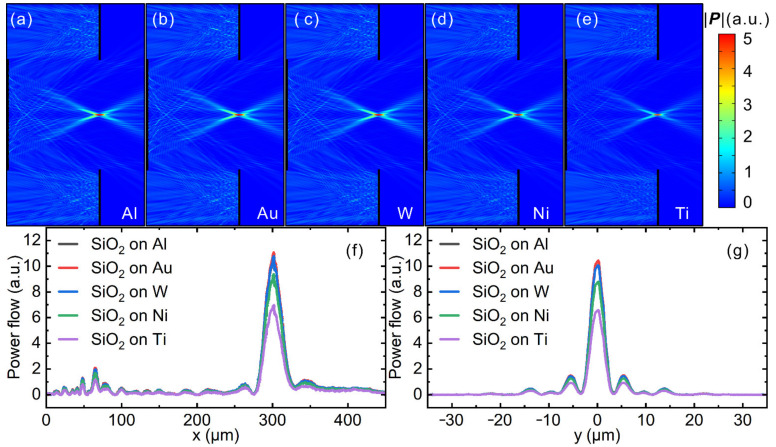
Optical performance of metasurface Cassegrain telescopes with metal constituents of (**a**) Al, (**b**) Au, (**c**) W, (**d**) Ti and (**e**) Ni, respectively, while all other parameters are the same as those discussed in the main text. Also shown are line-cut plots of the energy flux at the focus along (**f**) x- and (**g**) y-direction, respectively.

**Figure A2:**
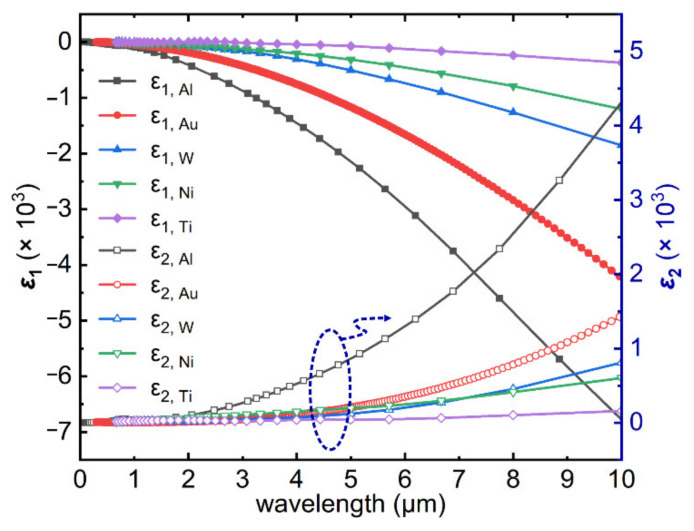
Real (*ε*_1_) and imaginary (*ε*_2_) parts of dielectric constants of various metals commonly used to construct nanophotonic devices in the deep-UV to mid-IR wavelength range.

To construct metasurface devices that possess the potential for real-world applications, optical properties such as the optical loss at design wavelength and operation bandwidth as well as abundance/cost and CMOS-compatibility need to be considered. Figure A2 in Appendix B shows dielectric constants from the deep-ultraviolet (DUV) to mid-infrared wavelength range (0.2–10 μm) of various metal materials commonly used to construct nanophotonic devices, including Al [31], Au [32], W [33], Ni [33] and Ti [33]. In our previous work, we demonstrated that the SiO_2_ plus Al material combination possesses the potential to construct metasurface devices covering a broad wavelength range from DUV to MWIR [24]. To further justify the choice of the SiO_2_ plus Al combination over others, we discuss below the optical performance of metasurface Cassegrain telescopes constructed with other materials. Metasurface Cassegrain telescopes with different metal constituents were constructed by replacing Al with Au, W, Ti and Ni, respectively, while keeping all other parameters the same as those discussed in Figure 3. It can be found in Figure 4 that at the design wavelength of 4 μm, all metasurface Cassegrain telescopes demonstrated a clear focus at the designed focal position. However, the peak intensity of the focus was different for different metal constituents. Devices with Al and Au demonstrated similar optical performance, both of which were better than the other three, i.e., W, Ni and Ti. This means that Al and Au have smaller optical loss in the infrared wavelength range than the other three kinds of CMOS-compatible metals (W, Ni and Ti). Au is also a good plasmonic metal in the infrared wavelength region that possesses low optical loss; however, in the visible and UV wavelength region (<500 nm), Al outperforms Au in the sense of smaller optical loss. This is because Au has inter-band transitions starting from 500 nm, and photons with higher energies will be absorbed by Au. Moreover, Au is a noble metal and much more expensive than Al. In addition, Au is not compatible with current CMOS technology, while Al is. Therefore, with comprehensive consideration of the operation bandwidth, optical loss, cost and CMOS-compatibility, we chose Al as the metal constituent to construct the metasurface device proposed in this work.

**Figure 5:**
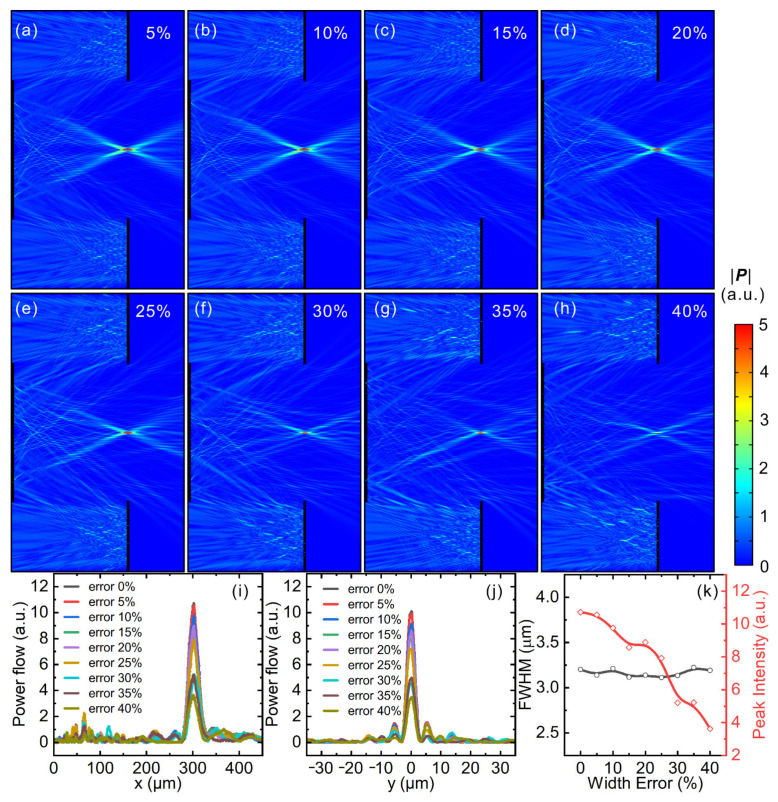
Energy flux of a metasurface Cassegrain telescope with f-ratio of 5/12 with (**a**) 5%, (**b**) 10%, (**c**) 15%, (**d**) 20%, (**e**) 25%, (**f**) 30%, (**g**) 35% and (**h**) 40% random width errors applied to the meta-atoms. Also shown are line-cut plots of the energy flux at the focus along (**i**) x- and (**j**) y-directions, as well as (**k**) FWHM (black-curve) and peak intensity (red-curve) of the focus for different devices with various levels of random width error of meta-atoms.

**Figure A5:**
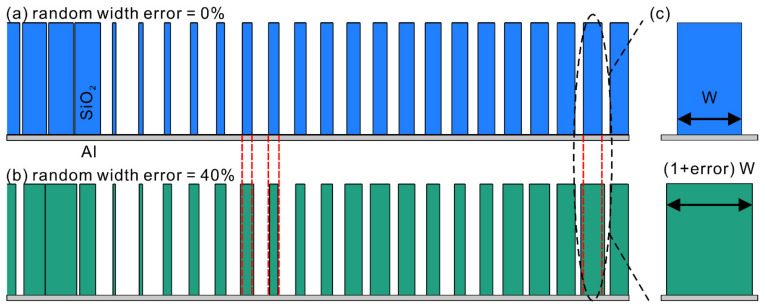
Schematic illustration of two metasurfaces with (**a**) 0% and (**b**) 40% random width error. (**c**) An enlarged view to show the different width of SiO_2_ stripes that correspond to W and (1 + *error*) W, respectively.

In practical applications, fabrication errors (deviation of the actual structures from the ideal version) are an important factor that influences the performance of metasurface devices, which is also an important issue in the field of optical engineering. For traditional Cassegrain telescopes based on curved mirrors, it is known that surfaces with ideal profiles provide the best optical performance; fluctuation/deviation in surface curvature usually reduces the optical performance of the whole system, which is a challenging issue encountered by astronomical telescopes with large apertures in particular. Great effort is continuously being devoted to minimize the fluctuation of surface curvature, which is inevitable even with state-of-the-art fabrication techniques, rendering manufacturing of large-scale curved mirrors quite costly and time consuming. On the contrary, in the case of flat optical devices based on metasurfaces, arrays of meta-atoms are lithographically fabricated in a way very similar to the manufacture of microelectronic chips [34]. In principle, the height of meta-atoms can be uniform, which is possible with current thin-film growth/etching technology. However, the widths of meta-atoms can deviate from the desired values during the fabrication process. In the following, we numerically studied the influence of width deviation on the overall performance of the metasurface Cassegrain telescope. To mimic fabrication errors, we purposely introduced certain random width errors to each meta-atom, while maintaining their height to be correct and uniform. A detailed description of the definition of random width errors and the way to apply them can be found in Appendix E. As can be seen in Figure A5a in Appendix E, meta-atoms with correct widths were placed at their design positions, while those with 40% random width error for each meta-atom were placed at corresponding positions with perturbed widths (Figure A5b). In this way, we constructed two sets of metasurface Cassegrain telescopes with two different f-ratios (5/12 and 5/6), where different levels (5~40%) of random width errors were applied to the meta-atoms. Simulation results for the optical performance of the Cassegrain telescope with an f-ratio of 5/12 are plotted in Figure 5a–h, which has the same design parameters as those discussed in Figure 3. Simulation results of the other set of Cassegrain telescopes with an f-ratio of 5/6 are displayed and discussed in Appendix C.

Figure 5a–h depicts the energy flux of metasurface Cassegrain telescopes with 5 to 40% random width error applied to the meta-atoms, whose design parameters are the same as those of Figure 3a. With the increasing level of random width error, it was found that the optical performance gradually deteriorated in the sense that the focus became dimmer and dimmer. Note that all subplots in Figure 5a–h share the same color bar on the right. Moreover, if one looks closely at the subplots, it can be noticed that energy flux toward the focus becomes more and more asymmetric, proving that the perturbed widths of meta-atoms negatively affect the optical performance of the whole system. To quantitatively evaluate the influence of width errors, we plotted in Figure 5i,j line-cut plots of the focus along the x- and y-directions, respectively. Figure 5k depicts the FWHM and peak intensity of various devices with different levels of random width error. It can be observed that the FWHM of different devices did not vary too much, whereas the peak intensity of energy flux at the focus noticeably dropped with increasing width error. The FWHM stayed roughly constant around an average value of 3.1 μm. The intensity at the focus achieved a value of 10.72 (arbitrary unit, a.u.) without width error and decreased continuously with increasing width error. If we define an intensity drop of 10%, 20% and 50% of the original value to be unacceptable, then the allowed random width error could be 10%, 15% and 25%, respectively. Modern optical systems based on curved mirrors with high precision usually require the surface fluctuation to be on the level of 0.1% [4,5], which is quite challenging for modern milling and polishing techniques, especially when the operational wavelength becomes short.

On the contrary, flat optical components based on metasurfaces can be fabricated with modern semiconductor manufacturing technology, such as lithography, deposition and etching. With state-of-the-art semiconductor manufacturing technology, the height of nano-/micro-structures can be kept uniform, and the lateral size deviation of structures can be minimized through optimizing the lithography layout and process. Optical lithography, such as deep ultraviolet (DUV) or extreme ultraviolet (EUV) lithography, is generally employed in semiconductor manufacturing industries. Random width errors brought about by the optical lithography method are mainly due to the proximity effect during the exposure process, which can distort the fine features of a metasurface. However, the proximity effect can be reduced or compensated with a method called computational lithography [35]—a specific research field dealing with the proximity effect. Furthermore, state-of-the-art nano-/micro-fabrication technology has made great progress, especially those with electron beam or ion beam methods [36]. Metasurface devices fabricated using electron beam lithography have shown quite high uniformity, and the accuracy in width can be better than 10 nm [8]. For the meta-atoms designed in this work, since the widths of the SiO_2_ stripes had a magnitude of several hundred nanometers, the width range and accuracy were within the fabrication capability of modern nanofabrication techniques. Thus, a width error of 25% is quite large for modern lithography and etching process, and much smaller width errors can be achieved using state-of-the-art planar fabrication technology. This implies that Cassegrain telescopes based on metasurfaces possess higher tolerance with respect to fabrication errors compared to traditional ones with curved surfaces. Of course, a more systematic study is required to conclude whether metasurface optical components in general possess higher tolerance with respect to fabrication errors compared to traditional ones; however, this is beyond the scope of the present work.

## 4. Conclusions

To summarize, in this work, we have proposed and numerically studied metasurface Cassegrain telescopes based on SiO_2_ plus Al meta-atoms. The SiO_2_ plus Al meta-atoms of suitable geometric size offer overall reflectance higher than 95% and a complete phase coverage of 0~2π at the operational wavelength of 4 μm. Several miniaturized Cassegrain telescopes were constructed and studied numerically, and the functionality of the Cassegrain telescope was confirmed by simulation results. The obtained focus had a lateral dimension of 3 μm—smaller than the operational wavelength. Moreover, the influence of fabrication errors was studied numerically by introducing random size errors to the meta-atoms. With increasing error levels, the optical performance gradually deteriorates. Error levels of 10%, 15% and 25% are estimated to be allowable if we define intensity drops of 10%, 20% and 50%, respectively, to be unacceptable. This work provides another notable design for Cassegrain telescopes based on metasurfaces that can be extended to other wavelength ranges, such as DUV. The estimation of fabrication errors can also provide some guidance to evaluate the optical performance of metasurface devices before launching large-scale fabrications.

## Figures and Tables

**Figure A3:**
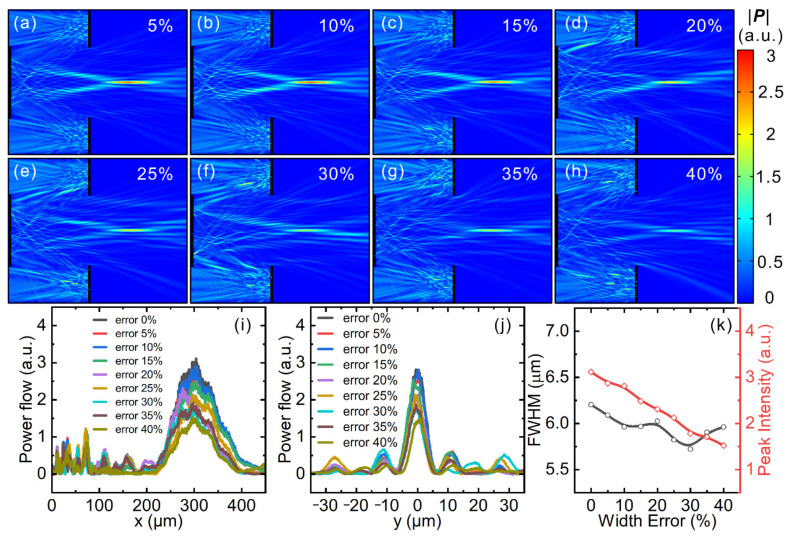
Energy flux of a metasurface Cassegrain telescope with f-ratio of 5/6 with (**a**) 5%, (**b**) 10%, (**c**) 15%, (**d**) 20%, (**e**) 25%, (**f**) 30%, (**g**) 35% and (**h**) 40% random width error applied to the meta-atoms. Also shown are line-cut plots of the focus along (**i**) x- and (**j**) y-directions, as well as (**k**) FWHM (black-curve) and peak intensity (red-curve) of the focus for different devices with various level of random width error of meta-atoms.

**Figure A4:**
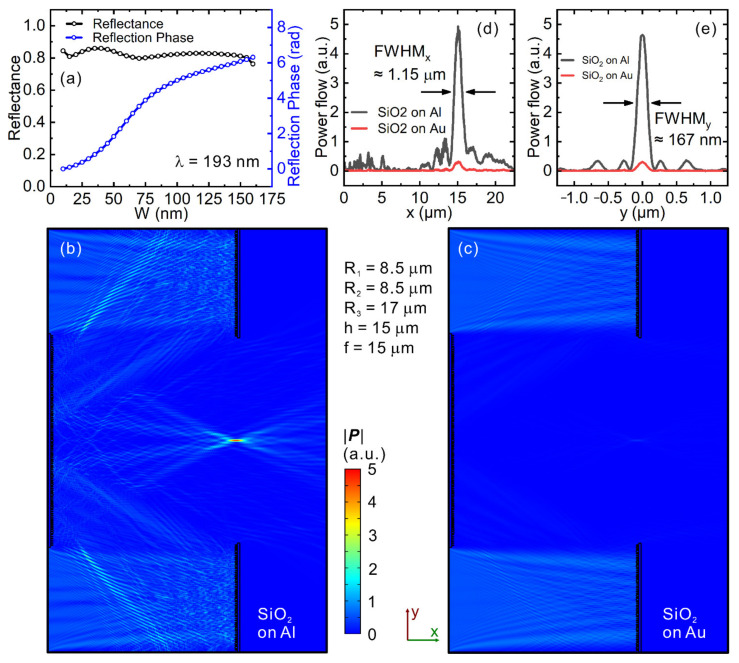
(**a**) Reflectance and reflection phases of the meta-atom unit cells with period P = 170 nm, height H = 190 nm and various widths (W). (**b**) Color map of energy flux of a metasurface Cassegrain telescope working at 193 nm wavelength with SiO_2_ plus Al as the constituent material. The design parameters are R_1_ = R_2_ = 8.5 μm, R_3_ = 17 μm and h = f = 15 μm. (**c**) The same as (**b**) except that Al is replaced by Au while keeping all others unchanged. Note that (**b**,**c**) share the same color bar in the middle. Also shown are line-cut plots of the focus along (**d**) x- and (**e**) y-directions, respectively. It is obvious that Al outperforms Au in UV wavelength range in the sense of smaller optical loss and, thus, greater intensity.

## Appendix A

### Typical Phase Distribution on the Primary and Secondary Mirror

The required phase distribution on the primary and secondary mirrors has been calculated using Formulas (1) and (2) in the main text and displayed in Figure A1. The metasurface Cassegrain telescope had the following design parameters: R_1_ = R_2_ = 180 μm, R_3_ = 360 μm, h = f = 300 μm. At each position represented by a circle on the phase profile, the required phase change is matched with the phase change provided by meta-atoms, and the matched meta-atoms are placed at each corresponding position. In this way, a metasurface layout can be obtained.

## Appendix B

### Comparison of Dielectric Constants of Different Materials

Figure A2 shows dielectric constants from the deep-ultraviolet (DUV) to mid-infrared wavelength range (0.2–10 μm) of various metal materials commonly used to construct nanophotonic devices, including Al [31], Au [32], W [33], Ni [33] and Ti [33]. As seen from Figure A2, at the design wavelength of 4 μm, the real part of the dielectric constants of all the abovementioned metal constituents is negative, meaning that they all demonstrate metallic properties at this wavelength. In this sense, all the abovementioned metal constituents can be considered in principle. However, the absolute value of the real part of the dielectric constant of Al is the greatest, indicating that it behaves more like a perfect electric conductor at the design wavelength compared with others. Other CMOS-compatible metals such as W, Ni and Ti are generally considered to be poor plasmonic metals due to their high optical loss. Au is also a good plasmonic metal in the infrared wavelength region that possesses low optical loss; however, in the visible and UV wavelength region (<500 nm), Al outperforms Au in the sense of smaller optical loss. This is because Au has inter-band transitions starting from 500 nm, and photons with higher energy will be absorbed by Au.

## Appendix C

### Optical Performance of Cassegrain Telescope System with Different f-Ratios

Based on the versatile capability of metasurfaces to manipulate light, Cassegrain telescopes with other design parameters (and thus different f-ratios) can also be realized. In particular, the focus can be set behind the exit pupil of the primary mirror—the same as the case of traditional Cassegrain telescopes. In addition, the aperture size of the metasurface Cassegrain telescope can also be varied. In the main text, the design parameters of the Cassegrain telescope are R_1_ = R_2_ = 180 μm, R_3_ = 360 μm and h = f = 300 μm, which gives an f-ratio of 5/12 (f-ratio = f/2R_3_). Here, the design parameters of the Cassegrain telescope are R_1_ = R_2_ = 90 μm, R_3_ = 180 μm, h = 200 μm and f = 300 μm, which gives an f-ratio of 5/6. With increasing levels of random width error applied to the meta-atoms, the optical performance of the metasurface Cassegrain telescope gradually deteriorates as well (Figure A3a–h). As seen from Figure A3k, it can be noticed that the most obvious difference between the two sets of devices is the FWHM of the focus. For the device with an f-ratio of 5/12, the FWHM of the focus was around 3.1 μm, although there was some fluctuation of the FWHM with different error levels for the width. For the device with an f-ratio of 5/6, the FWHM of the focus was around 6.0 μm—nearly twice the size of the focus of the other device. For both devices, the peak intensity dropped continuously with increasing width error. In addition, due to the smaller aperture of the device with an f-ratio of 5/6, the absolute value of peak intensity at the focus was smaller compared to the other device with an f-ratio of 5/12. From the comparison, we can see that although the optical performance of different devices is different, the trend and influence of random size error on the optical performance of metasurface devices is the same, i.e., with increasing width error, the FWHM of the focus stays roughly constant within a certain range while the peak intensity drops continuously. In addition, the energy flux towards the focus became more and more asymmetric with increasing error level.

## Appendix D

### Cassegrain Telescope Systems Working in the DUV Wavelength Range

In addition, since the SiO_2_ plus Al material combination possesses the potential to work from the deep-ultraviolet (DUV) to the mid-wave infrared (MWIR) range [24], and the metasurface Cassegrain telescope demonstrated in the main text was designed for an operational wavelength of 4 μm, it is worthy to see the performance of the metasurface device working in the DUV wavelength range, e.g., 193 nm. Here, we repeated the working procedure in the main text to find the optimal geometries of the meta-atom unit cells, and then constructed metasurface devices based on these unit cells. Figure A4a depicts the reflectance and reflection phases of meta-atoms working at 193 nm, with period P = 170 nm, height H = 190 nm and various widths (W). For all these meta-atoms, reflectance stayed higher than 80%, and the phase change range covered 0~2π, satisfying the selection criterion. Based on these meta-atoms, an exemplary metasurface Cassegrain telescope working at 193 nm was constructed, whose design parameters were R_1_ = R_2_ = 8.5 μm, R_3_ = 17 μm and h = f = 15 μm. A simulated color map and streamline plots of energy flux of the metasurface Cassegrain telescope are displayed in Figure A4b, were a clear focus can be observed at the exit pupil of the primary mirror. Line-cut plots of the focus (black curves in Figure A4d,e) show that FWHM values along the x- and y-directions are 1.15 μm and 167 nm, respectively. Considering that the operational wavelength was 193 nm, this metasurface Cassegrain telescope also achieved sub-wavelength focusing. If we replace Al with Au and construct the same metasurface device, we can obtain the optical performance in Figure A4c as well as the red curves in Figure A4d,e. It is obvious that Al outperforms Au in the UV wavelength range in the sense of smaller optical loss.

## Appendix E

### Metasurface Cassegrain Telescope with/without Random Size Errors

A metasurface device was constructed by putting corresponding meta-atoms at the correct positions; arrays of such meta-atoms constitute a metasurface. To mimic and evaluate fabrication errors of a metasurface on its optical performance, we introduced certain random width errors to each meta-atom, while keeping their positions and heights correct. To do so, we introduced a parameter, error, to represent fabrication errors applied to the widths of the SiO_2_ stripes. *Error* is a uniformly distributed random number with different magnitudes. If the correct width of a certain SiO_2_ stripe is W, then the perturbed width of the corresponding stripe is (1 + error) × W. For example, when the magnitude of *error* is 0.2, then error is a uniformly distributed random number in the range of [−0.2, 0.2], so the perturbed width of the SiO_2_ stripes is within the range of [0.8, 1.2] × W. When we constructed the metasurface device in our simulations, we could modify the width of the SiO_2_ stripes into (1 + error) × W and put them at the required positions one-by-one in a cycled way with a MatLab script. In this way, each meta-atom consisted of an SiO_2_ stripe with the above-defined random width error applied. Figure A5 gives an example of two metasurfaces with 0% and 40% random width error. Cassegrain telescopes with the same design parameters were constructed using metasurfaces with different levels of width error; their optical performances were compared in the main text.
